# Medical Advertisements through Dispensaries, Etc.

**Published:** 1877-12

**Authors:** 

**Affiliations:** Uptonville


### Correspondence.

UPTONVILLE, Sept. 9th, 1877.

*Editors Medical Journal:*

I have been pleased to see that my former letters have been read by some of your subscribers, and that they have attracted some attention. Their object seems however, to be misapprehended, I am not like the critics whom Disraeli hits so hard in his novel Lothair: one who unsuccessful in my calling try to cast mud at all others who follow the same pursuit. While I criticise I do so in the kindest spirit, I have not it is true been eminently successful, although to relieve those who give this or that one the credit of writing these letters I will confess that the pen portrait in the first letter was not taken from nature but may represent any of a large number of struggling young doctors.

The entire object of these missives is to show some of the obstacles which these young physicians meet in their endeavors to practice medicine honestly. Those obstacles are numerous, and all are not beset alike by them. Some may settle in a town where prescribing druggists abound, others where those who should be guides are led into by and forbidden paths whose intricate mazes are but dimly lit by moonshine, others may fall in with a class of older and well meaning fellow practitioners who think they are doing them a special favor when they speak of them in the presence of the laity as "Young" Doctor So and So, and reassuringly say to their patients when called in consultation:—"well your Doctor is doing very well, very well indeed, to be sure he is young, but he has at least done no harm, and *after this* I think he may perhaps change his treatment somewhat and you will do nicely, of course I will run in any time he desires."

So you see what I might be fighting against may not trouble Dr. C., and what troubles Dr. C., may have nothing to do in hindering the advance of either Dr. D., or myself. I have endeavored therefore to keep my eyes open. and by observation and conversation with my medical brethren keep informed as to the stumbling blocks in the way of the young man who follows medicine as a profession.

Young men when they receive their degree and are sent forth to cope with the world for an existence are taught with much reiteration the binding force of the code upon all honorable men, and many of them come to believe that they must fail to gain a practice and even endure the pangs of starvation before breaking one of its precepts. Having a brotherly feeling for all such, let me. show them how the code may be "honorably"(?) ignored, or at least, how those who would resent being called dishonorable, manage to evade its plain directions. There are, here in Uptonville, several dispensaries, or infirmaries, or call them what you will. There are the Free General Dispensary where all diseases both medical and surgicial in character are treated; the Provident Surgical Infirmary ; the Infirmary for Infants and Children ; the Women's Free Dispensing Union for diseases peculiar to females ; the Opthalmological Infirmary ( a name which at least attracts attention); the Uptonville Association for the relief of the Infirm Poor; and a Homœpathic institution of whose general character I am not informed. The ostensible object of these institutions is one that is most worthy and above reproach.

The poor we have always with us, and to lessen the burdens which they bear is a christian and benevolent act. But is this solely and purely the object of these Infirmaries, Dispensaries and like Associations? Those who are connected with them with seeming honesty and fairness will tell you that there is with them a secondary object in view. They desire to gain experience and practice in certain special lines and at the Dispensaries and Infirmaries they can separate these diseases into classes and study them as they desire. Ignoring, for the time at least, the question which some impertinently inquisitive patron of the Dispensaries may ask "should not these Doctors have perfected themselves in these lines before they commenced to "practice" on us?" let us see if this is the sole object in organizing a Dispensary. Young Doctor Wisehead receives his diploma and comes to Uptonville to establish himself he selects an office swings out his sign and awaits patients. From loyalty to the sublime teachings of the code or from fear of incurring the displeasure of his seniors he is deterred from announcing to the public that he gives special attention to diseases of women, or to surgery, or to diseases of the nervous system. Our young friend however has a keen eye to the main chances and accordingly looks about him, becomes suddenly interested in the down-trodden poor and connects himself with one of the Dispensaries intended to relieve their physical ills and in due time the public are informed in the annual report of the institution published in the Uptonville *Avalanch*, that Dr. Wisehead has charge of the department of nervous diseases, Dr. Jones of the department of surgery, Dr. Smith that of gynæcology and so on through the list, which is, as you see, a very ready and efficient means of advertising without paying the usual charges or seeming to break the code. The effect of this ready system of treating the poor, upon the pocket and practice of Dr. Wisehead and his brethren both in and out of dispensary and infirmary circles will be the theme of my next letter, my remarks will be based upon actual experience.

Yours very truly,

NIL DESPERANDUM.

